# Biomass-Based Silicon and Carbon for Lithium-Ion Battery Anodes

**DOI:** 10.3389/fchem.2022.882081

**Published:** 2022-05-04

**Authors:** Manoj Muraleedharan Pillai, Nathiya Kalidas, Xiuyun Zhao, Vesa-Pekka Lehto

**Affiliations:** Department of Applied Physics, University of Eastern Finland, Kuopio, Finland

**Keywords:** biomass, silicon, carbon, anode, lithium-ion battery

## Abstract

Lithium-ion batteries (LIBs) are the most preferred energy storage devices today for many high-performance applications. Recently, concerns about global warming and climate change have increased the need and requirements for LIBs used in electric vehicles, and thus more advanced technologies and materials are urgently needed. Among the anode materials under development, silicon (Si) has been considered the most promising anode candidate for the next generation LIBs to replace the widely used graphite. Si cannot be used as such as the electrode of LIB, and thus, carbon is commonly used to realize the applicability of Si in LIBs. Typically, this means forming a-Si/carbon composite (Si/C). One of the main challenges in the industrial development of high-performance LIBs is to exploit low-cost, environmentally benign, sustainable, and renewable chemicals and materials. In this regard, bio-based Si and carbon are favorable to address the challenge assuming that the performance of the LIB anode is not compromised. The present review paper focuses on the development of Si and carbon anodes derived from various types of biogenic sources, particularly from plant-derived biomass resources. An overview of the biomass precursors, process/extraction methods for producing Si and carbon, the critical physicochemical properties influencing the lithium storage in LIBs, and how they affect the electrochemical performance are highlighted. The review paper also discusses the current research challenges and prospects of biomass-derived materials in developing advanced battery materials.

## Graphical Abstract

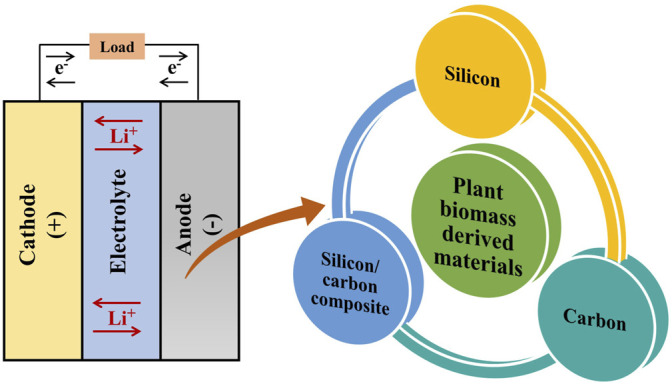

## 1 Introduction

The contributive capacity of secure and green energy in the growing economy and modern technology has increased the significance of electrochemical energy storage devices now more than ever (Yang et al., 2018). Among the various storage devices, LIBs demonstrate the highest potential and performance capacity (Zhao and Lehto, 2021). This increases the significance of research in developing different electrode materials that improve the performance of LIBs. The ethical and environmental values emphasize the need to derive such electrode materials from renewable sources such as agricultural residues. The development of electrode materials from agricultural residues ensures that energy production is economical, sustainable, and secure (Abbasi and Abbasi, 2010). This aspect enhances the significance of Li-battery production in promoting green energy.

In commercial LIBs, graphite is used as the anode due to its good electrical conductivity and excellent cycling stability, which offers a theoretical capacity of 372 mAhg^−1^. Typically, graphite is either mined or artificially manufactured using petroleum coke feedstock. As a result, it is a critical and expensive raw material (Dühnen et al., 2020). Recently, biomass-derived carbon materials have attracted substantial interest as anode materials for LIBs because of their favorable properties, including easy availability, sustainable production, low-cost and environmental friendliness. To date, the carbonaceous materials derived from various biomasses have been investigated as anodes in LIBs (Olsson et al., 2022). Biomass-derived carbon materials feature large specific surface areas and tunable porous structures that enhance ion transfer and diffusion. However, to fulfill the present energy demand, new anode materials with higher capacity are required for advanced LIBs, as the graphitic anode has reached its theoretical limit in commercial batteries (Lee et al., 2016). Owing to its high theoretical specific capacity (3,579 mAhg^−1^), nontoxicity, natural availability, and low working (0.4 V vs. Li/Li^+^) potential, Silicon (Si) is considered the most preferred choice for next-generation LIBs. However, the enormous volume change (300%) causes the electrode pulverization and rapid capacity fading of Si anode during the lithiation-delithiation process, which is a major drawback (Ashuri et al., 2016). To date, substantial research has been conducted to overcome the physical strain in Si anode by modifying Si into nanowires (Chan et al., 2008), nanopillars (Lee et al., 2011), and nanotubes (Wen et al., 2013). However, the synthesis approaches to obtain nano-Si anodes are often high-energy-consuming and sensitive, limiting their scalability and practical use. In this regard, developing Si materials from biogenic silica (SiO_2_) sources is an attractive and cost-effective alternative.

**FIGURE 1:**
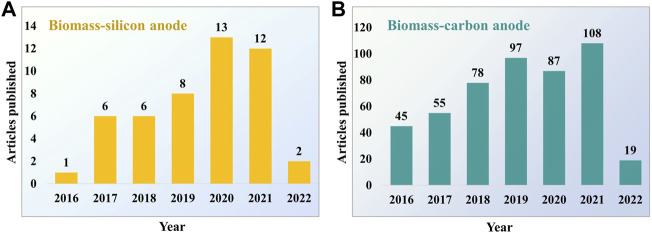
Number of publications on biomass-derived materials as anodes for LIBs from 2016 to March 2022. **(A)** Biomass-silicon anodes and **(B)** biomass-carbon anodes. Data from Web of Science by searching “biomass silicon anode” or “biomass carbon anode” and “lithium-ion battery”.

The growing interest in utilizing the biomass-derived materials as anodes in LIBs is visible in the increasing number of publications over the last few years (Figure 1). The present review paper focuses on the recent progress of plant biomass-derived Si and carbon anodes for LIBs. The details about various biomass precursors utilized, Si and carbon extraction methods, and the effect of their morphology impacting the electrochemical performance are discussed. Finally, the crucial obstacles related to applying biogenic derived materials in LIBs are described, and potential future research directions are envisaged.

## 2 Biomass-Derived Silicon for Lithium-Ion Batteries

Nanostructured Si is produced from agricultural residues simply and inexpensively. The agriculture residues are rich in phytoliths deposited as amorphous SiO_2_, which can be used as a precursor to synthesize Si. Therefore, the SiO_2_ structures are extracted from residues by acid purification and calcination processes. This SiO_2_ can be converted to Si through a reduction process so that the nanostructures can be preserved in the synthesized Si powder. The affordable biomass-derived Si can provide benefits in the development of next-generation LIBs.

### 2.1 Purification of Si Materials From Biogenic Silica From Different Bio-Sources

Agricultural residues are cheap, natural, renewable, and environmentally friendly sources to extract SiO_2_ for LIBs. The methods for extracting biobased SiO_2_ from agricultural wastes are subjected to extensive research. Various sources have been utilized for SiO_2_ extraction, such as rice husks (Zhang et al., 2016) (Kim et al., 2017), barley husk ash (Kalidas et al., 2020), bamboo leaves (Wang et al., 2015) (Lin et al., 2016), bamboo charcoal (Zhang et al., 2018), reed plants (Wang J. et al., 2021) (Liu et al., 2015), horsetail (He et al., 2018), sugarcane (Chen et al., 2021), and corn leaves (Su et al., 2020). The SiO_2_ content of the biosources mainly depends on the geological origin of the plants. Extraction of SiO_2_ from biosources mainly involves the leaching process and thermal treatment.

The leaching method is an efficient way to purify agricultural waste using strong acids. The agricultural waste raw material consists of organic components and alkali impurities. SiO_2_ is the major constituent in the residues of agricultural plants, along with various metal oxide content such as Al_2_O_3_, Fe_2_O_3_, MnO, ZnO, CuO, Sr_2_O_3_, TiO_2_, K_2_O, CaO, MgO, Na_2_O (Setiawan and Chiang, 2021). The initial step in the leaching method is removing the impurities. Hydrochloric acid (HCl) is identified as the most used acid for the purification process, as reported in studies (Zhang et al., 2016) (Kalidas et al., 2020) (He et al., 2018). The agricultural residues are pulverized using the ball milling method and then mixed with 10% HCl solution for 2–12 h at 70–120°C and are stirred to remove metallic impurities. Finally, the product is filtered and washed with deionized water, and later, the samples are dried at 60–120°C for 5–12 h (Wang Z. et al., 2021) (Wang et al., 2015) (Praneetha and Murugan, 2015) (Yu et al., 2018) (Ma et al., 2021).

In some agriculture residues, heat treatment is conducted, followed by a leaching process. After the leaching process, calcination is conducted to produce SiO_2_. Calcination is generally carried out at a temperature between 550 and 800°C for 2–6 h at a heating rate of 2–5°Cmin^−1^ in the air to produce SiO_2_ (Chen et al., 2021) (He et al., 2018). Depending upon the temperature, an amorphous or crystalline form of SiO_2_ can be obtained. The produced structures include SiO_2_ spherical nanoparticles with a diameter of about 40–90 nm (Sekar et al., 2019a) (Zhang et al., 2016), and nanoporous SiO_2_ with a surface area of 101–329 m^2^g^−1^ and pore diameter 3–8 nm (Liu et al., 2015) (Wang et al., 2015). The SiO_2_/C composites were produced from the acid leached agricultural waste annealed at a temperature of 400–900°C for 100 min to 3 h in an argon atmosphere (Lin et al., 2016) (Ma et al., 2021). For SiO_2_/C composite produced, rice husks have the lowest surface area of 18 m^2^g^−1^ (Yu et al., 2018), and sugarcane leaves have the largest surface area of 1,429 m^2^g^−1^ (Chen et al., 2021).

### 2.2 Reduction of Purified Si Materials From Biogenic Silica to Silicon

Metallothermic reduction is the most convenient way to convert metal oxides or non-metal oxides, sulfides, and halides into metals or non-metals, alloys, and composites. Metallothermic reaction is a self-propagating exothermic reaction using reactive metal as the reducing agent, including magnesium, lithium, sodium, aluminum, potassium, and calcium. The reducing metal should have strong reducing behavior, affordability, and easy melt. Additionally, its byproducts should be leached out simply from the metal product. For example, the metals (titanium, tantalum, niobium, and vanadium), non-metals (silicon and carbon), (Xing et al., 2016) (Xing et al., 2018), and composites (Li_2_S/transition metals (cobalt, nickel, zinc, iron, molybdenum, tungsten, titanium, manganese, copper)) (Xing et al., 2020) were produced by the metallothermic reduction process. The conventional method to synthesize Si from SiO_2_ is the carbothermal reduction method conducted with carbon in an electric arc furnace at a high temperature of 2000°C (Wang et al., 2015). Magnesium (Mg) or aluminum (Al) metal powder is often used to synthesize porous Si anodes as they have a solid reducing behavior and low melting point. In this process, SiO_2_ is mixed with Mg or Al in the different mass ratio (SiO_2_: Mg is 1: 0.8–1: 2.5, SiO_2_/C: Mg is 1: 0.2–1: 0.6 and SiO_2_/C: Al is 1:0.36–1:1) and the mixture is loaded into crucibles or Swagelok reactors. Magnesiothermic reduction is carried out at a temperature range of 650–750°C for 2–8 h with a heating rate of 2–5 and 10°Cmin^−1^ in nitrogen, argon, or argon/H_2_ atmosphere (Kim et al., 2017) (Zhang et al., 2018). For the aluminothermic reduction process, the temperature is 200–300°C for 14–18 h in an argon atmosphere (Chen et al., 2021) (Su et al., 2020). For the SiO_2_/C composite, the reduction temperature was 200 and 650–700°C for 2–6 h with a heating rate of 2–5°Cmin^−1^ under an argon atmosphere (Sekar et al., 2019a) (Lin et al., 2016). NaCl or AlCl_3_ molten salt that acts as a heat scavenger is often added to a mixture of SiO_2_ and Mg, absorbing the excess heat from the reaction. When the reduction temperature is close to the melting point of Mg at 650°C, the exothermic reaction is initiated, and the reaction releases heat. The reaction temperature continues to increase until 801°C, where NaCl salt starts to melt and absorbs the heat generated from the reaction. Therefore, the temperature is controlled by NaCl heat scavengers, which prevents the aggregation of the Si domain and the damage of pore structure (Luo et al., 2013).

The Mg or Al reacts with SiO_2_ and reduces it into Si according to the equation:

$$SiO_{2}\;+2Mg\to Si+2MgO$$ (1)

$$3SiO_{2}\;+4Al\to 3Si+2Al_{2}O_{3}$$ (2)

The obtained sample was soaked in 1–2 M HCl for 6–12 h to remove the byproducts of magnesium oxide or aluminum oxide. Finally, pure Si with porous structure is obtained with SiO_2_ impurity. The remaining SiO_2_ is dissolved by immersing the sample into 1M HF or 5% HF solution. The produced nanostructured Si has a lower surface area of 54–302 m^2^g^−1^, pore volume 0.30–0.73 cm^3^g^−1^, and pore diameter 0–6 nm (He et al., 2018) (Wang J. et al., 2021) (Wang et al., 2015) (Praneetha and Murugan, 2015). For Si/C, the surface area is 28–201 m^2^g^−1^, pore diameter 6–9 nm, pore volume 0.065–0.30 cm^3^g^−1^ (Liao et al., 2021) (Lin et al., 2016) (Yu et al., 2018). The magnesiothermic reduction conditions significantly affect the morphology, particle size, surface area, and pore volume of Si. The main advantages of the magnesiothermic reduction process are simplicity, slower reaction time, and low energy consumption. Therefore, the above-summarized material properties of synthesized Si are advantageous for high-performance LIB anode material.

### 2.3 Biomass-Derived Silicon-Based Anodes in Lithium-Ion Batteries

Generally, Si anodes have the issue of low cycling stability that hinders their application as the electrode in LIBs. The volume expansion issue in Si anodes during cycling can be efficiently addressed by reducing the size of Si, which gives extended cycle performance. Rice husks were used to produce porous Si nanoparticles with diameters ranging from 10 to 40 nm (Liu et al., 2013). Due to their small size and porous nature, the obtained nano-Si displayed a longer cycle life and better battery performance than commercial metallurgical-grade Si particles and commercial non-porous Si nanoparticles (Liu et al., 2013). Although Si nanoparticles have better cycle stability than their bulk counterparts, the intense solid-electrolyte interphase (SEI) layer formation and a low volumetric energy density restrict their practical application.

**FIGURE 2:**
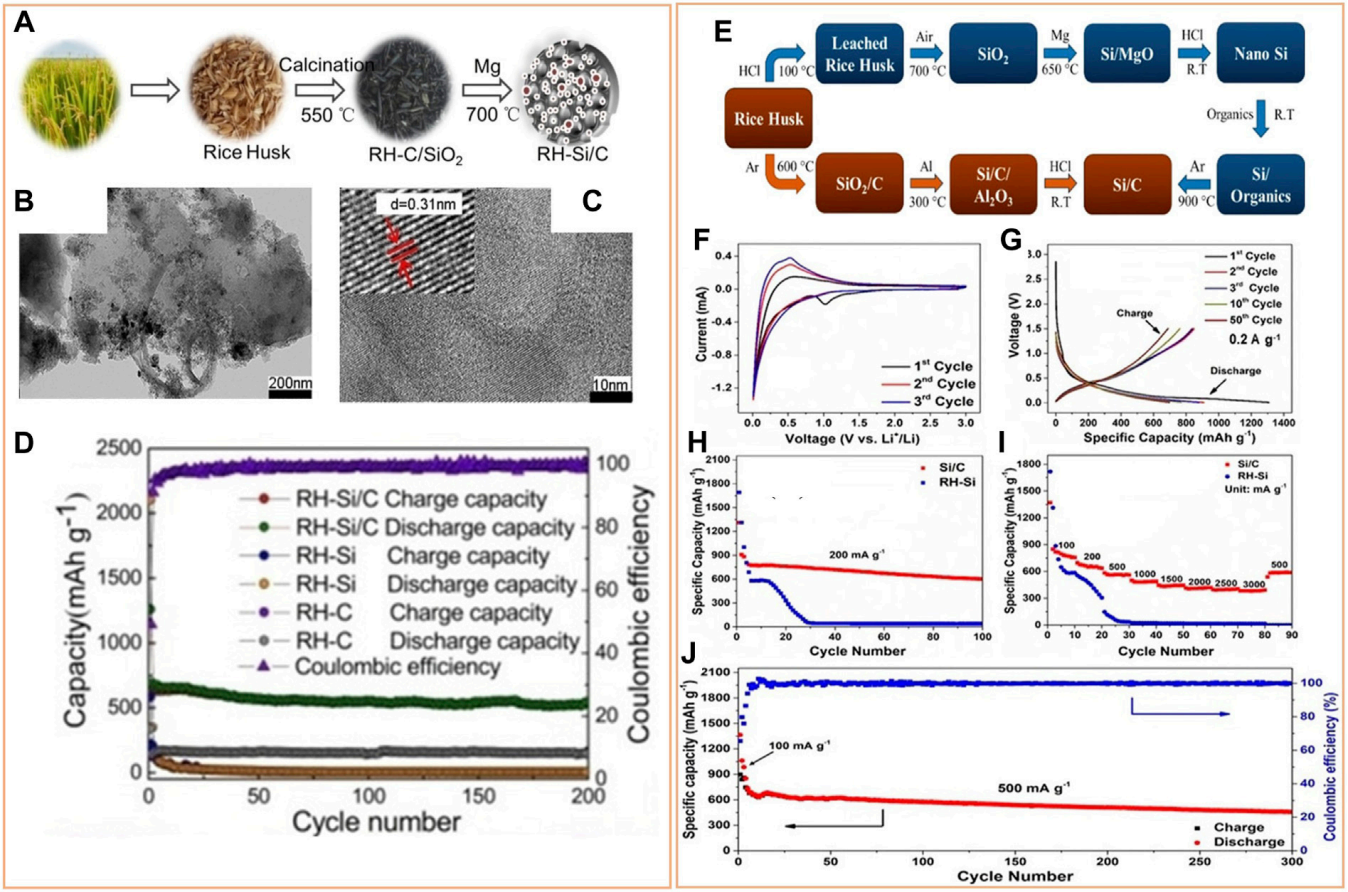
**(A)** Schematic representation of the synthesis of Si/C composite from RH *via* calcination and magnesiothermic reduction method. **(B)** TEM image of RH-derived carbon. **(C)** TEM images of RH-derived Si/C composite. **(D)** Cycle performance of RH-derived carbon, Si, and Si/C composite at a current density of 0.1 Ag^−1^. Modified with permission from (Yu et al., 2018). Copyright © 2000–2022 by John Wiley & Sons, Inc. **(E)** Schematic illustration of the preparation process for RH-derived Si/C using aluminothermic reduction. Electrochemical performance of Si/C composite: **(F)** CV curves, **(G)** charge/discharge curves, **(H)** cycling performance at 200 mAg^−1^, **(I)** rate capabilities at different current densities, and **(J)** cycling stability at 500 mAg^−1^. Modified with permission from (Majeed et al., 2020). Copyright © 2000–2022 by John Wiley & Sons, Inc.

Incorporating carbon-based materials to biogenic generated Si is preferred to alleviate the difficulty created by volume expansion since carbon materials can buffer the material dimension changes. Carbon can improve the electrical conductivity of the Si anode and create a conductive network facilitating fast lithium-ion transfer. Furthermore, the SEI layer formed on the Si/C electrode surface is stable, preventing further cracking, pulverization, and agglomeration of Si particles during cycling and maintaining structural stability. The carbon used in the Si/C composite can be from the Si source itself or external sources. Usually, organic polymers (Kummer et al., 2014) (Guo et al., 2005) and carbonaceous materials such as graphite (Su et al., 2014), graphene (Zhou et al., 2012), and carbon nanotubes (Zhang et al., 2014) serve as external carbon sources in Si/C composite. For instance, using a layer-by-layer assembly method, Wang et al. prepared a Si@C/RGO nanocomposite before producing Si nanoparticles (5–8 nm) from bamboo leaves by a magnesiothermic reduction process (Wang et al., 2015). The carbon shell and RGO provided double protection, minimizing volume fluctuations, avoiding direct contact between Si and the electrolyte, enhancing electrical conductivity, and eventually improving battery performance. Compared to an external carbon source, it is more economical and simpler to prepare Si/C composite anodes from the same biomass source. Yu et al. developed a robust porous 3D network Si/C composite anode using rice husk (RH) as the source of Si and carbon (Figure 2A). The porous RH-derived carbon (Figure 2B) acted as a buffer for Si volume expansion as well as a link between the isolated Si nanoparticles. The existence of crystalline Si nanoparticles in the carbon network was observed (Figure 2C) in the synthesized Si/C composite. This composite electrode displayed superior performance compared to RH-carbon and RH-Si (Figure 2D) (Yu et al., 2018). Majeed et al. also synthesized porous Si/C composite from RH by aluminothermic reduction at a lower temperature (Figure 2E), which exhibited good electrochemical performance (Figures 2F–J) compared to RH-Si (Majeed et al., 2020). Therefore, the development of Si/C anode from biomass raw materials appears to be the most reliable strategy for the future, considering its economic value and eco-friendly aspect. However, the innovative synthesis methods are still highly desirable as there is a clear gap between the current technology and practical industry application. In Table 1, various biomass-derived Si-based materials as anodes for LIBs are summarized.

**TABLE 1:** Biomass-derived Si-based anodes for LIBs.

Materials	Biomass source	Morphology	Surface areaof Si/C composite (m^2^g^−1^)	Electrochemical performance (ICE^a^ or CC^b^/DC^c^, cycling stability)	Ref.
Si/C	Rice husks	Nanoparticles	27.47	72%, 901.5 mAhg^−1^ at 100 mAg^−1^ after 50 cycles	Liao et al. (2021)
Si/C	Reed leaves	Hollow nanostructure	343.9	96%, 1,548 mAhg^−1^ at 100 mAg^−1^ and 650 mAhg^−1^ at 500 mAg^−1^ after 200 cycles	Wang et al. (2021a)
NPSi@C	Rice husks	Nanoporous	270.5	41%, 681.8 mAhg^−1^ at 0.2 Ag^−1^ after 100 cycles	Wang et al. (2021b)
Corn-Si	Corn Leaves	Porous and amorphous/crystalline mixed structure	56.1	-, 2,100 mAhg^−1^ at 0.5 Ag^−1^ after 300 cycles and 1,200 mAhg^−1^ at 8 Ag^−1^	Su et al. (2020)
Si/C	Rice husks	Nanoparticles	—	-, 90% capacity retention after 150 cycles at 0.5 C and a charge capacity of 420.7 mAhg^−1^ at 3 C	Ma et al. (2021)
Si/C	Rice husks	Mesoporous	—	65%, 460 mAhg^−1^ at 500 mAg^−1^ after 300 cycles	Majeed et al. (2020)
TCPSi/CNT-600–2	Barley husks	Mesoporous	102	-/1,213, 770 mAhg^−1^ at 0.2 C after 50 cycles	Kalidas et al. (2020)
AC < nc-Si > AC	Rice husks	Spherical nanoparticles (40–60 nm)	498.5	97.5%, 429 mAhg^−1^ at 200 mAg^−1^ after 100 cycles	Sekar et al. (2019a)
Si@N/C	Bamboo Charcoal	3D hierarchical porous structure	111.23	67.4%, 603 mAhg^−1^ at 200 mAg^−1^ after 120 cycles and 360 mAhg^−1^ at 1.6 Ag^−1^	Zhang et al. (2018)
Si@N-C	Horsetails	Nanoparticles	273.59	-/1,148.8, 1,047.1 mAhg^−1^ at 0.5 Ag^-1^ after 450 cycles and 750 mAhg^−1^ at 1 Ag^−1^ after 760 cycles	He et al. (2018)
Si/C	Rice husks	3D porous nanoparticles	199	49.18%, 537 mAhg^−1^ at 0.1 Ag^−1^ after 200 cycles	Yu et al. (2018)
c-Si_RH_-graphite (1:9)	Rice husks	Mesoporous	—	93.8%, 432.2 mAhg^−1^ at 1 C after 100 cycles	Kim et al. (2017)
Si@C	Bamboo leaves	Porous	201	1,080/1,648, 600 mAhg^−1^ at 2 Ag^−1^ after 3,700 cycles	Lin et al. (2016)
rGO-porous Si	Rice husks	Porous	239	68.8%, 830 mAhg^−1^ at 1 Ag^−1^ after 200 cycles	Jiao et al. (2016)
Si/N-C/CNT	Rice husks	Microsphere (3.2 µm)	78.5	72%, 1,031 mAhg^−1^ at 0.5 Ag^−1^ after 100 cycles	Zhang et al. (2016)
Si@C/RGO	Bamboo leaf	Nanoparticles (5–8 nm)	—	79%, 1,400 mAhg^−1^ at 2 C and 60% capacity retention on increasing C-rate from 0.2 to 4 C	Wang et al. (2015)
Si⊂C	Reed leaves	Highly porous 3D structure	224	2,435/4,000, 420 mAhg^−1^ at 10 C after 4,000 cycles	Liu et al. (2015)
Si/C	Rice husks	3D nanoporous	172 (Only Si)	77.5%, 1,997 mAhg^−1^ at C/5 after 200 cycles-, 1,290 mAhg^−1^ at C/5 after 200 cycles-, 1,166 mAhg^−1^ at C/5 after 200 cycles	Praneetha and Murugan, (2015)
Si/GNS	Rice husks	3D nanoporous			
Si/MWCNT	Rice husks	3D nanoporous			
Si nanoparticles	Rice husks	Porous nanoparticles (10–40 nm)	245	-/2,790, 86% capacity retention after 300 cycles	Liu et al. (2013)

a ICE: Initial Coulombic Efficiency.

b CC: Charge Capacity (mAhg^−1^).

c DC: Discharge Capacity (mAhg^−1^).

## 3 Biomass-Derived Carbon for Lithium-Ion Batteries

### 3.1 Precursors of Biomass-Derived Carbon

Plant-based biomass materials, renewable carbon-rich sources, offer the prospect of developing anode electrodes for LIBs as they have tunable surface properties and are readily available, low-cost, sustainable, and environmentally benign. These materials are mainly composed of cellulose, hemicellulose, and lignin, which serve as the biomass carbon source. Generally, crops and residues remain the primary source of biomass-derived carbon electrodes. Generally, carbon is the major constituent in biomass compositions with a minor content of oxygen, hydrogen, nitrogen, and sulfur. However, the exact composition of biomass precursors is influenced by the biomass species, growing habitat, geographical location, and seasonal changes. Various biomass precursors were investigated, including wheat bran (Wang et al., 2020) avocado seeds (Yokokura et al., 2020), reed flowers (Zhao et al., 2020), cherry pits (Hernández-Rentero et al., 2020), green-tea waste (Sankar et al., 2019), coffee grounds (Luna-Lama et al., 2019), peanut dregs (Yuan et al., 2019), loofah (Wu et al., 2019), jute fiber (Dou et al., 2019), coffee oil (Kim et al., 2018), corn stalks (Li et al., 2018), apple, celery (Hao et al., 2018), wheat flour (Lim et al., 2017), coir pith (Mullaivananathan et al., 2017), orange peel (Xiang et al., 2017), woodchips (Adams et al., 2016), prolifera-green-tide (Cui et al., 2016), wheat stalk (Zhou et al., 2016), coconut oil (Gaddam et al., 2016), rice (Han et al., 2016), and cotton (Zhu and Akiyama, 2016). Cotton, a viable raw material for carbon manufacturing, contains 90–95% cellulose, thus making it one of the most abundant and environmentally favorable biomass materials in nature.

### 3.2 Preparation Methods of Biomass-Derived Carbon

#### 3.2.1 Pyrolysis

Pyrolysis is the widely employed method to synthesize carbon materials from biomass. This method is the thermochemical conversion of organic materials without oxygen at elevated temperatures (<1,000°C). The biomass sources can yield carbon materials with different physical structures, depending on the pyrolysis conditions like temperature ramping rate, temperature, and time. The composition and structure of the precursor determine the properties of biomass-derived carbon materials, which often result in high surface areas, porosities, and distinctive morphologies. After pyrolysis, the carbon products require a chemical activation procedure to increase the surface area and obtain various pore size distributions. Activation is the most practical approach for increasing the surface area of carbon materials. The different chemical activation reagent used includes KOH (Yuan et al., 2019), KClO_4_ (Qiu et al., 2020), H_3_PO_4_ (Hernández-Rentero et al., 2020), CuCl_2_ (Dou et al., 2019), CaCl_2_ (Li et al., 2018), and ZnCl_2_ (Ru et al., 2016). Potassium hydroxide (KOH) is the most common activation agent used due to its low activation temperature. Additionally, the use of KOH results in a high yield of carbon products with enhanced specific surface area and porosity. Pyrolysis of orange peel at 800°C with and without KOH activation (OPDHC-A and OPDHC) was carried out by Xiang et al. An enhancement in the surface area was found for OPDHC-A (638 m^2^g^−1^) compared to OPDHC (357 m^2^g^−1^) (Xiang et al., 2017). However, activation can also reduce the electrical conductivity of carbon materials. Xing et al. conducted a study to understand the fundamental activation mechanism of amorphous carbon. In the study, CO_2_ was used to activate graphite, soft carbon, and hard carbon. They found that the electrical conductivity of all carbon structures decreased after activation as the entire carbon structure became hollow and damaged during the activation (Xing et al., 2017). Therefore, activation of carbon should be very carefully designed to obtain the carbon with an appropriate surface area and electrochemical conductivity.

#### 3.2.2 Hydrothermal Method

The hydrothermal method is another carbonization technique to synthesize carbon materials in an aqueous solution at moderately lower temperatures (<250°C) under high pressure in a sealed vessel. This approach allows very homogeneous products to be synthesized under comparatively moderate conditions. Compared with other synthesis methods, this method offers easy control of the surface chemistry and improvement in the electronic property of the resultant carbon by additional thermal treatment. Furthermore, the yield of the carbonaceous materials is high (>70%) compared to pyrolysis, and here, no drying process is required for biomass precursors as the process takes place in water. Without using any chemical activation reagents, Wang et al. performed hydrothermal carbonization to produce porous carbon materials from rice husk for Li-ion battery applications. Here, the cellulose component of rice husk was used as the precursor after removing lignin and hemicellulose fraction using formic acid. Hierarchical fibrous carbons with improved conductivity were obtained by subsequent calcination at 1,000°C and enhanced porosity after SiO_2_ removal with NH_4_HF_2_. In order to impart porosity into the resultant carbon material, the SiO_2_ component functioned here as an “*in situ*” hard template. The intermediate hydrothermal treatment improved the yield from 43 to 60% compared to direct calcination (Wang et al., 2013).

#### 3.2.3 Air Expansion Method

Compared to the conventional methods of direct carbonization and hydrothermal biomass materials, the air-expansion method fixes the issue of volume shrinking and facilitates the wide-scale preparation of carbonaceous aerogels using a simple and inexpensive method (Han et al., 2016). The Air-expansion method developed by Han et al. used non-porous rice as the biomass precursor to fabricate hierarchically macroporous, mesoporous, and microporous structured carbonaceous aerogels with a large specific surface area (461.6 m^2^g^−1^). Here, rice was pressurized to roughly 200 pounds per square inch after being subjected to a proper moisture level. The pressure held inside the kernel causes it to puff out when the pressure is suddenly released. Puffed rice with a porous, spongy texture was obtained due to the interaction between starch and moisture. Carbonaceous aerogels are produced in this subsequent high-temperature carbonization process. Because of its exceptional porosity architecture and N-doped structure, the carbonaceous aerogel developed in this method demonstrates outstanding electrochemical performance when employed as LIB anodes.

#### 3.2.4 Template Method

Template materials are incorporated into biomass precursors before thermal treatment to create necessary carbon structures. Using cotton cellulose as the biomass raw material and MgO as a template, Zhu et al. presented a unique and scalable procedure to fabricate porous carbon. When cotton was absorbed in Mg (NO_3_)_2_ solution, the MgO template was produced, which was inserted into the cotton-derived carbon. After acid leaching to remove the pore creator MgO template, the fabricated porous carbon exhibits a high specific surface area of 1,260 m^2^g^−1^ with interconnected microporous and mesoporous structures (Zhu and Akiyama, 2016).

#### 3.2.5 Dry-Autoclaving Method

The dry-autoclaving method has been reported to obtain micrometer-sized spheroidal carbon. It is an environmentally friendly, single-step, fast, solvent, and catalyst-free method. Dry autoclaving is a straightforward synthesis technique that increases the possibility of reproducibility to fabricate carbon particles for LIB anodes without any activation agents (Kim et al., 2018). At high temperatures, coffee oil as the raw material decomposes, and its subsequent carbon crystallization during cooling leads to the formation of dumbbell-shaped carbon particles with a surface area of 5 m^2^g^−1^.

In summary, pyrolysis carbonization, activation-related carbonization, and hydrothermal carbonization remain the most often employed techniques to produce bio-carbon anodes. Chemical activation is commonly used to produce biomass carbon materials with a high surface area, which results in significant porous structures. The enhanced porosity creates more channels to access electrolytes easily and transport lithium ions quickly. Different activation agents can significantly influence the structural, morphological characteristics of resultant carbon products, which ultimately affect the electrochemical performance of the product. This is achieved by increasing accessible active sites and providing short pathways for quick ion transfer. However, activation can result in low yield and irregular-shaped or microporous carbon structures, unfavorable for electrolyte ion diffusion and transfer. Hence, it is desirable to optimize the kind, amount of activation agent, and activation temperature to obtain high-performance carbon anodes.

### 3.3 Biomass-Derived Carbon Anodes in Lithium-Ion Batteries

The critical factors that improve the electrochemical performance of carbonaceous material in LIBs are (1) High specific surface area which provides more electrochemically active sites, resulting in a better electrode-electrolyte interface, speeding up charge transfer, and promoting Li^+^ adsorption, resulting in increased capacity; (2) Fast ion diffusion obtained by designing hierarchical porous structures resulting in improved rate capability; (3) Increase in the degree of graphitization which facilitates greater ion intercalation improving the electrochemical activity; (4) Doping of carbon anodes with heteroatoms causing defects, improving active site accessibility, and modifying electrical and chemical characteristics effectively, resulting in higher electrochemical reactivity in LIBs.

Designing carbon materials with porous structure and higher surface area can significantly improve their electrochemical performance in LIBs. The porous carbon with a high specific area of 1,260 m^2^g^−1^ was fabricated utilizing MgO as a hard template and cotton cellulose as a precursor (Zhu and Akiyama, 2016). When used as an anode, the porous carbon offered an initial Coulombic efficiency of 45.95% and exhibited good rate capability. The highly porous structure was beneficial in shortening lithium-ion diffusion length, increasing the electrode-electrolyte contact area, and mitigating the volume expansion issue during the lithiation process. Interconnected highly graphitic carbon nanosheets (HGCNS) have been successfully produced from the wheat stalk by Zhou et al. in a combined hydrothermal and graphitization method. The obtained mesopore structured HGCNS featured graphite-like interlayer spacing (0.34 nm) with a high degree of graphitization (90.2%) after the thermal treatment at 2,600°C (Zhou et al., 2016). These distinct characteristics of HGCNS facilitated multiple sites for the storage and insertion of Li ions and the fast mass movement of electrons and Li-ions. When utilized as an anode material for LIBs, HGCNS displayed an initial Coulombic efficiency of 63.2% at 0.1 C, excellent rate capability, improved cycling stability with a significantly decreased charge-discharge voltage hysteresis. Mesoporous dominant graphene-like carbon materials with a lattice spacing of 0.383 nm and a high degree of graphitization was prepared from peanut dregs by pre-carbonization and KOH activation followed by pyrolysis at 900°C. The obtained carbon material exhibited a low initial Coulombic efficiency of 36.2% when used as an anode material for LIBs. The presence of mesopores and a high degree of graphitization allowed rapid ion transfer and increased ion intercalation, benefiting improved battery performance (Yuan et al., 2019). Table 2 lists various plant-derived biomass carbon materials employed for LIBs, focusing on non-doped carbon anodes.

**TABLE 2:** Biomass-derived non-doped carbon anodes for LIBs.

Biomass source	Synthesis method	Morphology	Surface area (m^2^g^−1^)	Electrochemical performance (ICE^a^ or CC^b^/DC^c^, cycling stability)	Ref.
Spruce wood	Pyrolysis and H_3_PO_4_ activation	Hard carbon with micro and mesopores	61	65%, 300 mAhg^−1^ at 0.1 C after 400 cycles	Drews et al. (2021)
Wheat Bran	Carbonization	Honeycomb-shaped porous structure	57	85%, 515 mAhg^−1^ at 0.5 Ag^−1^ after 1,000 cycles	Wang et al. (2020)
Avocado seeds	Pyrolysis	Non-graphitic carbon	—	>90%, 315 mAhg^−1^ at 100 mAg^−1^ after 100 cycles	Yokokura et al. (2020)
Reed Flowers	Hydrothermal and multistep calcination	Hierarchically porous carbon with defects	1,715	61.1%, 581.2 mAhg^−1^ at 100 mAg^−1^ after 100 cycles and 298.5 mAhg^−1^ at 1,000 mAg^−1^ after 1,000 cycles	Zhao et al. (2020)
Hemp stems	Carbonization and KClO_4_ activation	Mesopore dominant hierarchical porous carbon	735	54.65%, 1,030 mAhg^−1^ at 0.1 Ag^−1^ after 100 cycles and 346 mAhg^−1^ at 5 Ag^−1^ after 2,000 cycles	Qiu et al. (2020)
Cherry pits	Annealing and H_3_PO_4_ activation	Highly disordered carbons with micropores and mesopores	1,662	<50%, 170 mAhg^−1^ at C/3 after 100 cycles	Hernández-Rentero et al. (2020)
Green tea powder	Air-assisted carbonization and KOH activation	Mesoporous graphitic carbon nanoflakes (6–10 nm)	1,373	64.4%, 400 mAhg^−1^ at 0.1 Ag^−1^ after 100 cycles	Sekar et al. (2019b)
Green tea wastes	Carbonization and KOH activation	Spherical mesoporous nanoparticles (30 nm)	1,241	55%, 498 mAhg^−1^ at 0.1 Ag^−1^ after 100 cycles	Sankar et al. (2019)
Coffee grounds	Carbonization	Non-porous and disordered stacked carbon	10	-1,764, 220 mAhg^−1^ at 0.1 Ag^−1^ after 100 cycles	Luna-Lama et al. (2019)
Peanut dregs	Carbonization, KOH activation and graphitization	Mesopores dominant graphene-like structure	2,040	36.2%, 286 mAhg^−1^ at 1000 mAg^−1^ after 100 cycles	Yuan et al. (2019)
Loofah	Pyrolysis and KOH activation	Three dimensional porous carbon	270	225 mAhg^−1^ at 100 mAg^−1^ after 200 cycles	Wu et al. (2019)
Jute fiber	Carbonization and CuCl_2_ activation	Disordered porous carbon	2,043	1,095.9/1,794.6, 580 mAhg^−1^ at 0.2 C after 100 cycles	Dou et al. (2019)
Coffee oil	Dry autoclaving	Sphere shaped structure with mesopores	5	34.5%, 290 mAhg^−1^ at 100 mAg^−1^ after 200 cycles and 350 mAhg^−1^ at 100 ^−1^ after 200 cycles at 50°C	Kim et al. (2018)
Corn stalks	Carbonization and CaCl_2_ activation	Mesoporous structure with pore size around 9.65 nm	370	60.16%, 783 mAhg^−1^ at 0.2 C after 100 cycles	Li et al. (2018)
Apple fine fiber	Annealing	Hierarchically porous carbon	16	73%, 1,050 mAhg^−1^ at 0.1 Ag^−1^ after 200 cycles	Hao et al. (2018)
Wheat flour	Pyrolysis	Highly disordered carbons	262	405/728, 217 mAhg^−1^ at 1 C after 100 cycles	Lim et al. (2017)
Coir pith	Carbonization and KOH activation	Microporous carbon with pore size around 1.4–1.7 nm	2,500	44%, 837 mAhg^−1^ at 100 mAg^−1^ after 50 cycles	Mullaivananathan et al. (2017)
Orange peel	Pyrolysis and KOH activation	Microporous structure with pore size around 0.7 nm	638	40%, 301 mAhg^−1^ at 1 Ag^−1^ after 100 cycles	Xiang et al. (2017)
Woodchip	Pyrolysis and KOH activation	3D structure with amorphous carbon sheets	1,580	49%, 650 mAhg^−1^ at C/5 after 250 cycles	Adams et al. (2016)
Prolifera green tide	Pyrolysis and KOH activation	Multilevel hierarchical porous carbon having microtubular morphology (30–50 µm)	2,200	39.9%, 523 mAhg^−1^ at 0.5 Ag^−1^ after 300 cycles	Cui et al. (2016)
Wheat stalk	Hydrothermal and graphitization	Graphitic carbon nanosheets with mesopores	35.5	63.2%, 139.6 mAhg^−1^ at 10 C after 3,000 cycles	Zhou et al. (2016)
Cotton cellulose	Template assisted carbonization	Disordered carbon with interconnected macro-mesopores	1,260	45.95%, 793 mAhg^−1^ at 0.5 Ag^−1^ after 500 cycles and 355 mAhg^−1^ at 4 Ag^−1^	Zhu and Akiyama, (2016)
Coconut oil	Incineration and piranha treatment	Quasi-spherical morphology	133	55%, 577 mAhg^−1^ after 20 cycles	Gaddam et al. (2016)
Bean dregs	Pyrolysis and graphitization	Ordered graphitic carbon	—	60%, 396 mAhg^−1^ at 0.1 C after 100 cycles	Ru et al. (2016)
Rice	Air expansion method	Hierarchically porous carbonaceous aerogels	461.61	63.6%, 505 mAhg^−1^ at 0.1 C after 110 cycles	Han et al. (2016)
Peanut shell	Pyrolysis and KOH activation	3D microporous carbon	706.1	523/1,077, 474 mAhg^−1^ at 1 Ag^−1^ after 400 cycles and 310 mAhg^−1^ at 5 Ag^−1^ after 10,000 cycles	Lv et al. (2015)
Corn starch	Carbonization	Sphere shaped structure having macropores	559	65.7%, 507 mAhg^−1^ at 0.1 Ag^−1^ after 100 cycles	Chen et al. (2015)

a ICE: Initial Coulombic Efficiency.

b CC: Charge Capacity (mAhg^−1^).

c DC: Discharge Capacity (mAhg^−1^).

In general, carbon materials derived from biomass precursors are a type of hard carbon which lacks crystalline characteristics and has low electrical conductivity compared to graphitic carbon. Even though a high specific surface area of carbon anode enhances the capacity, it results in low initial Coulombic efficiency due to the large lithium consumption to form the SEI layer. Hence, weaker electrical conductivity, largely irreversible capacity, and continuous voltage hysteresis during battery cycling are downsides of biomass-derived carbon. Additionally, high-temperature pyrolysis is often required to create a well-developed graphitic structure of materials, which is not in line with low-energy consumption and a low-cost production route. Further, it is difficult to get a high graphitization degree and nanostructures design for biomass-derived carbons limiting their commercialization for LIBs.

## 4 Conclusion and Prospects

The investigation of biomass resources to fabricate electrode materials for rechargeable batteries is prompted by the desire to develop eco-friendly materials and green energy storage devices. Biomass-derived materials have gained widespread attention and have great potential for developing anodes for LIBs. They are beneficial in promoting sustainability and circular economy. This review summarized the study of various biomass-derived Si and carbon anodes for LIBs, including their synthesis processes, morphological features, and electrochemical performance.

With the current scenario of energy requirements, the conventional graphitic anode must be replaced by a high-capacity Si anode to increase the energy content in LIBs. However, poor cycle stability and the formation of an unstable SEI layer due to volume changes upon cycling hinder the development of Si anodes. Although porous structured Si derived from a biogenic source can address the issue, it comes at the expense of volumetric energy density, which is vital for practical applications. The most common method for creating a stable Si anode is to combine Si with a conducting matrix, where carbon remains the most popular choice. Despite various studies that have been conducted to fabricate a Si/C composite anode, an ideal Si/C nanocomposite has not been developed yet. In addition, investigations on using Si and carbon from the same biomass sources to develop Si/C composites in a single step are limited. This method is more appealing due to its cost-effectiveness and green approach than conventual Si/C fabrication techniques, which are expensive and require substantial investment in equipment. The above-stated approach can ensure effective conversion of biomass to active materials, and the fabricated composite has a high potential for recycling. Hence, more attention should be placed on economic and scalable manufacturing processes to fabricate biomass-derived Si/C anode and commercialize these materials. Additionally, the full cell results of biogenic derived Si-based materials would be important in real-world applications, but those are seldom reported in studies. Hence, greater emphasis should be given to studying real battery performance rather than half-cell studies.

Despite numerous attempts to extract carbon from various biomass sources, the strategies for biomass selection and biomass conversion efficiency are rarely mentioned in reports. Inhomogeneity of the source material from year to year and habitat to habitat as well as its availability depending on climatic conditions can emerge as issues. As the selection of precursor materials can significantly influence the properties of the synthesized carbon material, a selection criterion needs to be established. The difficulty of purifying the bio-based material to obtain high-quality nanomaterial affordably should also be considered. A better knowledge of the composition of biomass precursors is required in future research. Biomass-derived carbons are favored as promising anode material for lithium-ion batteries because of their low cost and can be synthesized with green and straightforward methods. However, specific issues limit their commercial application as biomass-derived carbons typically display low initial Coulombic efficiency and rate capability compared to commercial graphite. The hetero-atom doping is an efficient approach to create synergetic effects to enhance the electrochemical performance of bioderived carbons by promoting fast electron transport and reducing the lithium diffusion path. Despite these promising strategies, if the bio-derived materials are to be commercially used, the performance of the LIB should not be compromised, and the expense of these materials should not be higher than that of the present materials.
